# New experimental melting properties as access for predicting amino-acid solubility†

**DOI:** 10.1039/c8ra00334c

**Published:** 2018-02-08

**Authors:** Yeong Zen Chua, Hoang Tam Do, Christoph Schick, Dzmitry Zaitsau, Christoph Held

**Affiliations:** Institute of Physics, University of Rostock Albert-Einstein-Str. 23-24 18051 Rostock Germany; Competence Centre CALOR, Faculty of Interdisciplinary Research, University of Rostock Albert-Einstein-Str. 25 18051 Rostock Germany; Laboratory of Thermodynamics, Department of Biochemical and Chemical Engineering, Technische Universität Dortmund Emil-Figge-Str. 70 44227 Dortmund Germany christoph.held@tu-dortmund.de; Chemical Institute A.M. Butlerov, Kazan Federal University 18 Kremlyovskaya Street Kazan 420008 Russian Federation; Institute of Chemistry, University of Rostock Dr-Lorenz-Weg 2 18051 Rostock Germany

## Abstract

The properties of melting are required for the prediction of solubility of solid compounds. Unfortunately, direct determination of the enthalpy of fusion and melting temperature by using conventional DSC or adiabatic calorimetry is often not possible for biological compounds due to decomposition during the measurement. To overcome this, fast scanning calorimetry (FSC) with scanning rates up to 2 × 10^4^ K s^−1^ was used in this work to measure the melting parameters for l-alanine and glycine. The enthalpy of fusion and melting temperature (extrapolated to zero heating rate) were Δ_fus_*H* = (22 ± 5) kJ mol^−1^ and *T*_fus_ = (608 ± 9) K for l-alanine, and Δ_fus_*H* = (21 ± 4) kJ mol^−1^ and *T*_fus_ = (569 ± 7) K for glycine. These melting properties were used in the modeling framework PC-SAFT to predict amino-acid solubility in water. The pure-component PC-SAFT parameters and one binary parameter were taken from literature, in which these parameters were fitted to solubility-independent thermodynamic properties such as osmotic coefficients or mixture densities. It was shown that this allowed accurately predicting amino-acid solubility in water over a broad temperature range. The combined methodology of PC-SAFT and FSC proposed in this work opens the door for predicting solubility of molecules that decompose before melting.

## Abstract 2

New experimental melting properties combined with PC-SAFT allow quantitative solubility predictions of amino acids in water.

## Graphical abstract

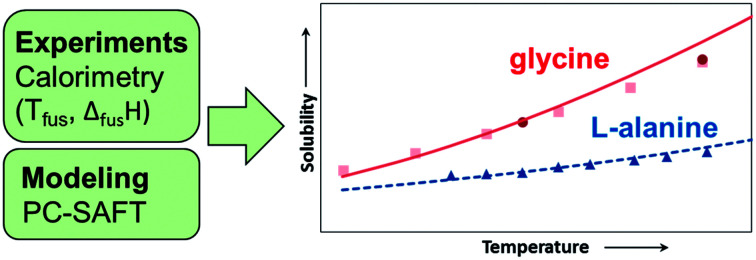

## Introduction

For the production and purification of amino acids, crystallization is the state of the art unit operation. The solubility of amino acids plays an essential role for crystallization as solubility determines the supersaturation level, product yield and purity as well as the choice of solvent for the process.^1^ Contrariwise (bio)chemical processes require knowledge about solution conditions that allow avoiding amino-acid precipitation. To quantify these conditions, the solubilities of the amino acids must be known. The experimental measurement of such solubilities is in general time-consuming and expensive, especially based on the almost innumerable different conditions in biological solutions which influence the solubility of biomolecules (temperature, pH-value, type and concentration of co-solutes and co-solvents). The prediction of solubility using thermodynamic models is therefore strongly desired. Such model predictions are possible using an equilibrium condition between the liquid and the solid phase. Assuming no mixed solids (pure solid amino-acid phase) and neglecting the influence of different heat capacities of solid and liquid amino acid, the mole fraction of the amino acid in the liquid phase (its solubility, *x*^L,sat^_*i*_) can be calculated according ref. 2 by1
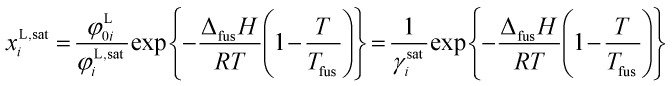
where *γ*^sat^_*i*_ is the activity coefficient of component *i* at its solubility and *T*_fus_ and Δ_fus_*H* are melting temperature and molar enthalpy of fusion, respectively. The activity coefficient is expressed as the ratio of fugacity coefficients of component *i* in its pure-component state and at the solubility mole fraction is accessible by thermodynamic models. For the thermodynamic modelling of amino acid solutions, different types of models have been reported so far, activity-coefficient models and equations of state. Xu *et al.*^3^ used the modified Wilson model with two adjustable parameters per system to calculate the activity coefficients in polymer aqueous solutions and the solubility of amino acids in aqueous solutions. It was possible to obtain better predictions of the solubility for higher temperatures compared to UNIFAC (Universal Quasichemical Functional Group Activity Coefficients) and UNIQUAC (Universal Quasichemical). Pazuki *et al.* published different models using three-parameter model based on the perturbation theory^4^ and M-Wilson, M-NRTL^5^ to model the activity coefficients of amino acids and simple peptides in water. Besides activity-coefficient models also equations of state have been applied to model amino-acid solutions. Mortazavi-Manesh *et al.*^6^ used a two-parameter model based on the perturbation of a hard-sphere reference to predict activity coefficients in aqueous solutions of amino acids. Later, Ji and Feng^7^ modeled activity coefficients of amino acids in water and aqueous solutions by using statistical associating fluid theory (SAFT). Ferreira *et al.*^8^ used perturbed-chain SAFT (PC-SAFT) for the prediction of several aqueous alkanol solution containing amino acids. Hereby the amino acids were treated as non-associating molecules. In contrast, Held *et al.* explicitly accounted for association forces in amino-acid solutions for PC-SAFT modeling.^9^ The results for modeled thermodynamic properties are still outstanding. Recently, Valavi *et al.*^10^ used perturbed hard sphere chain (PHSC) equation of state for the thermodynamic modeling amino acids and peptides in aqueous solutions. The amino acid molecules were treated as associating components with two association sites per each molecule. It is possible to predict some thermodynamic data as well as the solubility of binary and mixed amino acid and peptide aqueous solutions.

As further observed in eqn (1), solubility modeling requires experimental melting properties (*T*_fus_ and Δ_fus_*H*). However, experimental data for these melting properties have been inaccessible until now for substances that underlie thermal decomposition prior to melting (*e.g.* amino acids). The state of the art for solubility modeling of these substances is simultaneous adjustment of model parameters (in order to quantify *φ*_0*i*_*/φ*_*i*_) and melting properties based on experimentally determined solubilities. That is, the melting properties have been used as fit parameters. As the fitted melting properties depend on the accuracy and reliability of the applied model, different melting properties of amino acids have been obtained. This shortcoming complicates transferability and hinders acceptance of thermodynamic models for amino-acid solutions in industry. In order to overcome this, precise experimental melting properties are required. Such data will be presented in the present work.

The experimental data for l-alanine and glycine were measured using fast scanning calorimetry (FSC) which avoids thermal decomposition before and during melting, as has been successfully employed for melting of bio-polymers,^11,12^ for low molecular mass compounds^13^ and for the nucleobase cytosine.^14^ In the present paper, the melting parameters of pure amino acids have been successfully measured. The melting temperatures for l-alanine and glycine are (608 ± 9) K and (569 ± 7) K respectively, while the enthalpies of fusion are (22 ± 5) kJ mol^−1^ and (21 ± 4) kJ mol^−1^, respectively.

The existence of new experimental melting properties opens the door for new solubility models and will increase accuracy of prediction results up to very high temperatures (below amino-acid melting temperature). Thus, the experimental melting properties accessed in this work will serve as an input to solubility predictions of amino acids in water. It should be noted that not only PC-SAFT but also other models can be used for such solubility predictions, and the combination of FSC with PC-SAFT is just one possibility.

## Experimental

### Materials and reagents

The commercial samples of amino acids l-alanine (Sigma-Aldrich, mass purity ≥99% stated by the manufacture, CAS: 56-41-7) and glycine (Sigma-Aldrich, ≥99%, CAS: 56-40-6) were used without further purification.

### Measurement of melting properties

The melting properties of l-alanine and glycine were characterized by Mettler Toledo Flash DSC1 (ref. 15) with thin film chip sensors USF1.^16^ The measurements were performed under an inert atmosphere of nitrogen with a flow rate of about 50 ml min^−1^. The empty sensor was conditioned according to manufacturer's procedure. In order to achieve high heating/cooling rates with fast scanning calorimetry, the sample must be small, less than 100 ng. However, the surface-to-volume ratio increases for such small sample, and this would create significant mass loss due to evaporation or sublimation at elevated temperatures.^14,17^ This mass loss was partially suppressed by coating the sample with silicon oil.

**Fig. 1:**
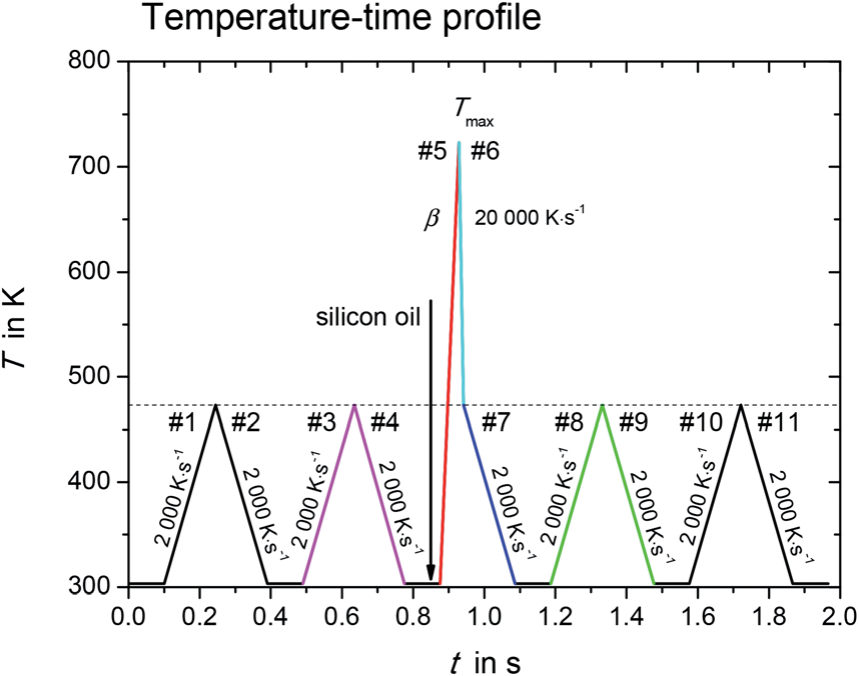
Temperature–time profile for determination of melting properties with fast scanning calorimetry. In heating step #5, the scanning rate, *β*, varied from 1000 K s^−1^ to 10 000 K s^−1^.

Fig. 1 shows the temperature–time profile used in this study. It is divided into three measurement stages: (i) sample mass determination (#1–#4); (ii) sample melting and quenching (#5–#7), and (iii) re-heating of supercooled sample (#8–#11).

During the first stage with heating/cooling cycles in scanning steps #1 to #4, the initial sample mass was determined and the sample was checked for absence of any mass loss due to volatile impurities. The sample without silicon oil was heated and cooled from temperature 303 K to 473 K at constant scanning rate *β*, *β* = 2000 K s^−1^. The results obtained in the heating/cooling cycles in scanning steps #1 to #4 were checked for reproducibility, indicating no mass loss in this temperature range and scanning rate. The initial sample mass (without silicon oil) was determined as

2 *m*_0_ = *C*_p_/*c*_p_

where *C*_p_ is heat capacity of the solid sample [J K^−1^], obtained from heating step #3 and cooling step #4 (magenta segments in Fig. 1) and *c*_p_ is specific heat capacity [J g^−1^ K^−1^]. The determination of sample mass is done according to ref. 14 and 18. As shown in ref. 18, the sample mass determination has an error of about 11% and contributes to the uncertainty of enthalpy of fusion determination. Specific heat capacity *c*_p_ for solid l-alanine and glycine was taken from the literature.^19,20^ An example of a measurement scan used for sample mass determination is shown in ESI, Section S1.†

Once the sample mass was known, the melting properties were determined in the second stage. After cooling step #4, the sample was coated with silicon oil. This reduces the surface of the sample exposed to the purge gas, and thus decreases the mass losses due to sublimation and evaporation drastically. Additionally, the silicon oil strongly increases the thermal contact between the sample and the sensor, which helps to avoid large thermal lag.

The sample with silicon oil was heated from 303 K to *T*_max_ during the heating step #5 (red segment in Fig. 1). The value for *T*_max_ is about 10 K to 20 K greater than the endset temperature of the melting peak, which increases with increasing heating rate. Too high overheating of the sample was avoided to prevent possible thermal decomposition and evaporation. The scanning rate of heating step #5 was varied from 1000 K s^−1^ to 10 000 K s^−1^. This allowed determining the true thermodynamic melting properties by extrapolating the measured properties to zero heating rate.

The time, at which the sample was kept at high temperatures, was kept as small as possible in order to minimize the sample mass loss due to evaporation. Thus, the sample was cooled rapidly down to 473 K at a programmed rate of 20 000 K s^−1^ (cooling step #6, cyan segment in Fig. 1) after melting. The ultra-fast quenching of the melted sample then allowed the sample to retain in the liquid state below the melting temperature (supercooled liquid). At 473 K the cooling rate was reduced to 2000 K s^−1^ (step #7, blue segment in Fig. 1). If crystallization in steps #6 and #7 could not be observed, the last stage with scanning steps #7 to #11 allowed investigating glass transition as well as possible low temperature crystallization of the sample.^14^ This will not be discussed in the present work.

### PC-SAFT equation of state

#### The model

Modeling solubility using eqn (1) requires amino-acid fugacity coefficients in its pure-component state and at the solubility mole fraction. Using SAFT-based equations of state, fugacity coefficients are expressed as3
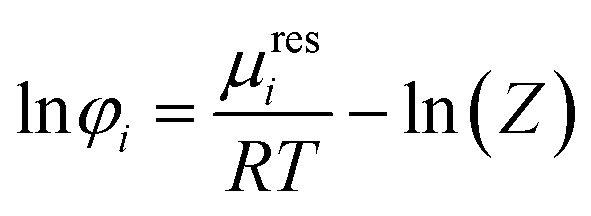
where *μ*^res^_*i*_ and *Z* are the residual chemical potential and the real gas factor, respectively. In order to calculate *μ*^res^_*i*_ and *Z*, the residual Helmholtz energy *a*^res^ is required. In this work, the following expression was used:

4 *a*^res^ = *a*^hc^ + *a*^disp^ + *a*^assoc^

where *a*^hc^, *a*^disp^ and *a*^assoc^ account for the Helmholtz-energy contributions due to hard-chain repulsion, dispersion and association interactions. All these contributions were used as in the original PC-SAFT model.^21^ To describe mixed solutions, the conventional Berthelot–Lorenz – combining rules were used for interactions between two components *i* and *j* (*e.g.* water and amino acid):5
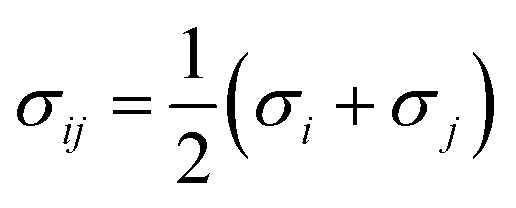
6
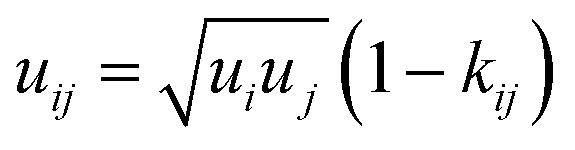


The binary interaction parameter *k*_*ij*_ is a fit parameter that describes deviations from the geometric mean of the dispersion–energy parameters of components *i* and *j*.

### PC-SAFT parameters

Water was modeled with 2B association scheme with temperature-dependent segment diameter as introduced by Cameretti *et al.*^22^ Both, the amino group as well as the carboxylic group of an amino acid were characterized with each one association site, *i.e.* amino acids in this work were modeled with 2B association scheme as well. The PC-SAFT pure-component parameters for the amino acids were taken from literature.^9^ As these were fitted to thermodynamic properties of aqueous solutions, these parameters depend on the used water parameters. Thus, the water parameters used in ref. 9 were also used in the present work. The PC-SAFT parameters used in this work are listed in Table 1.

**Table 1:** PC-SAFT parameters for glycine, l-alanine, and water used within this work and *k*_*ij*_ between amino acid and water. For all components, the 2B association scheme was applied

Component	*m* ^seg^_*i*_	*σ*_*i*_	*u*_*i*_ */k*_B_	*ε* ^A_*i*_B_*i*_^ */k*_B_	*κ* ^A_*i*_B_*i*_^	*k*_*ij*_ to water
l-alanine^9^	5.4647	2.5222	287.59	3176.60	0.0819	−0.0612^c^
glycine^9^	4.8495	2.3270	216.96	2598.06	0.0393	−0.0585^b^
water^22^	1.2047	a	353.94	2425.67	0.0451	—

a For water, a temperature-dependent segment diameter *σ* = 2.7927 + 10.11 exp(−0.01775*T*) − 1.417 exp(−0.01146*T*) was used.

b This work.

c A temperature-dependent *k*_*ij*_ was applied according to ref. 9: *k*_*ij*_(*T*) = −0.0612 + (*T* − 298.15)(2.91 × 10^−4^).

**Fig. 2:**
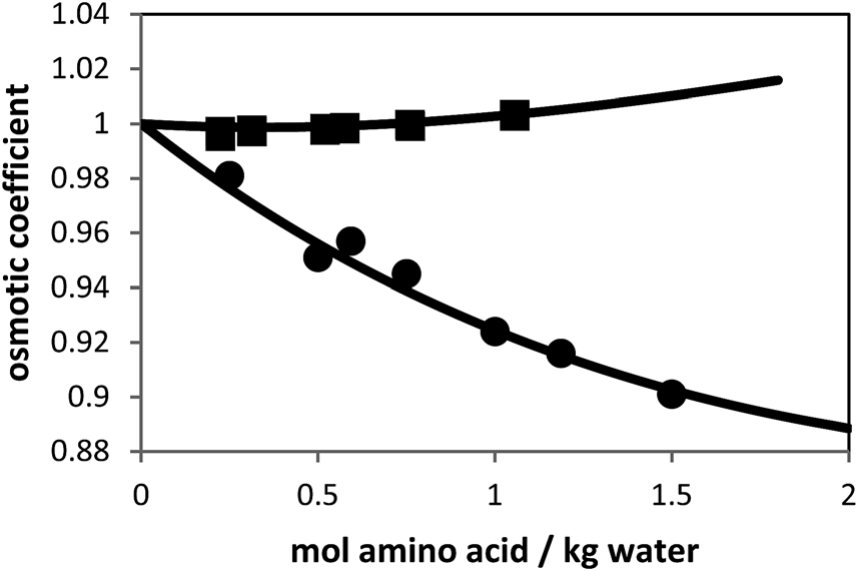
Osmotic coefficients of l-alanine + water and glycine + water solutions at 298.15 K. Symbols are experimental data (solid squares: l-alanine, and solid circles: glycine^9^) and lines are PC-SAFT modeling results with parameters from Table 1.

Besides the pure-component parameters, one binary interaction parameter was applied between amino acid and water according to eqn (6). In this work, the values for *k*_*ij*_ were fitted to experimental osmotic-coefficient data of amino-acid solutions at 298.15 K and atmospheric pressure. The result of the parameter fit can be observed in Fig. 2.

## Experimental results

### Melting temperature of the amino acids

The fast scanning calorimetry was used to characterize the melting temperature and enthalpy of fusion of l-alanine and glycine. The crystalline samples were measured over a range of scanning rates, *β*, from 1000 K s^−1^ to 10 000 K s^−1^.

**Fig. 3:**
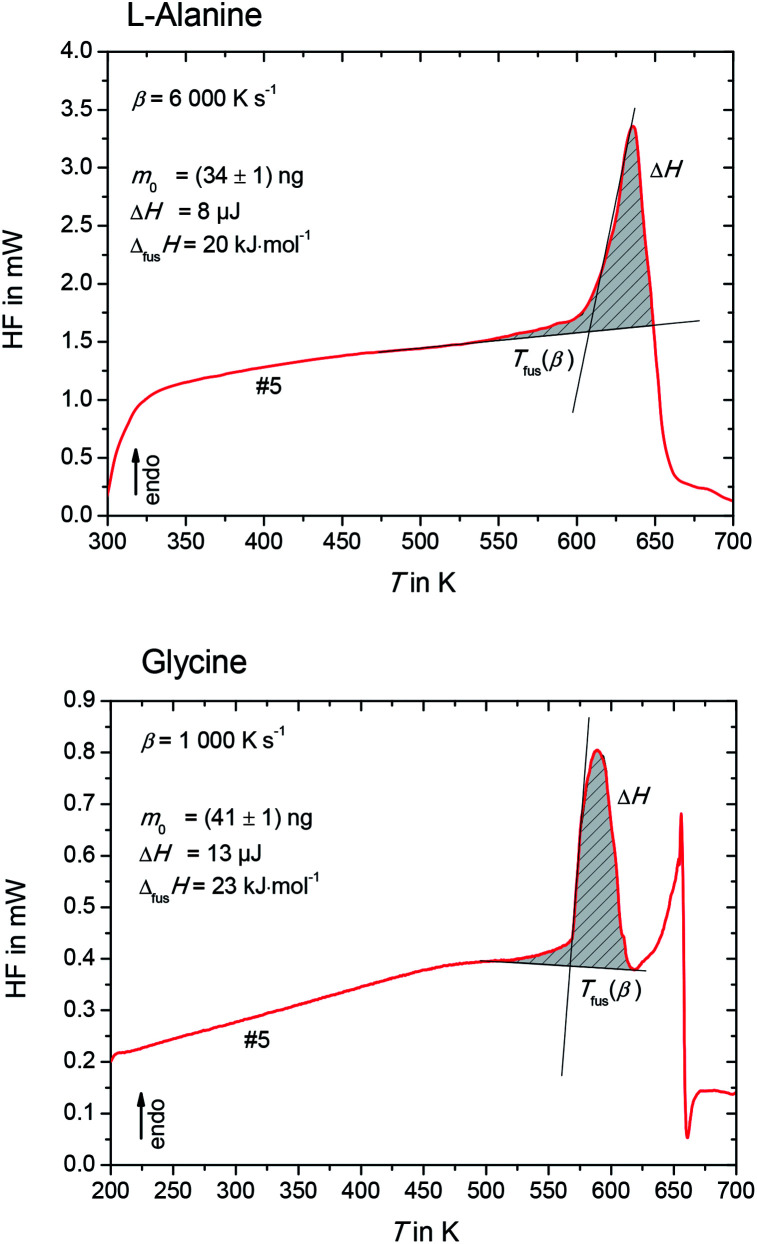
Heat flow rate curve of l-alanine (upper figure) and glycine (lower figure) in heating step #5. The melting temperature, *T*_fus_(*β*), is determined as the onset of the melting peak, while the enthalpy, Δ*H*, as area under curve.

The heat flow rate curves used for the determination of the apparent melting temperature, *T*_fus_(*β*) for l-alanine and glycine are shown in Fig. 3.

**Fig. 4:**
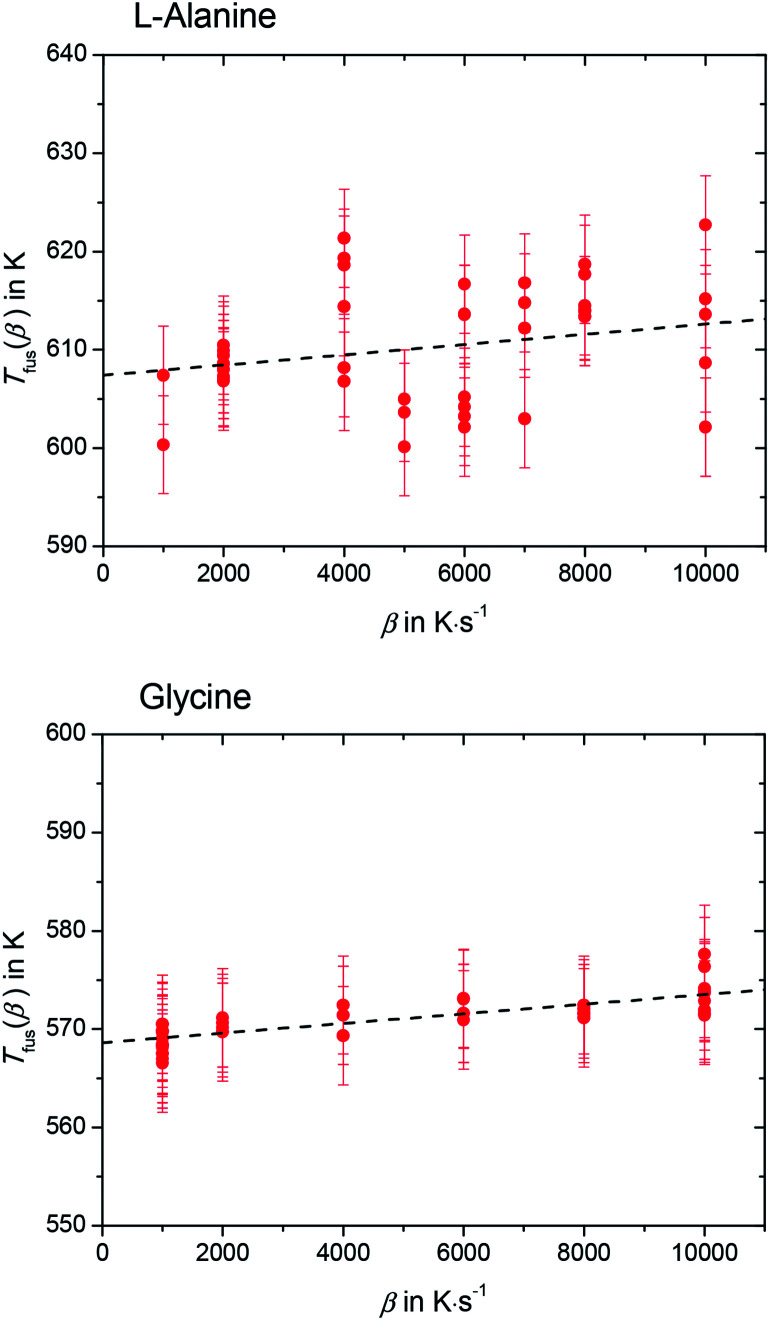
Extrapolated peak onset temperature of the melting peak of l-alanine (upper figure) and glycine (lower figure), as function of heating rate. The melting temperature at zero heating rate for l-alanine and glycine is *T*_fus_ = (608 ± 9) K and *T*_fus_ = (569 ± 7) K, respectively.

Please note that *T*_fus_(*β*) is not the thermodynamic fusion temperature as the sample was melted at very high heating rates. The presence of silicon oil could optimize thermal contact between sample and sensor, but a perfect heat transfer could not be obtained. Thus, a shift of *T*_fus_(*β*) with increasing *β* was observed. The value for the thermodynamic melting temperature *T*_fus_ is defined as the peak onset temperature measured for varying heating rates extrapolated to zero heating rate, *i.e. T*_fus_ = *T*_fus_(*β* → 0).^23^ To account for this phenomena, values for *T*_fus_(*β*) were plotted as a function of *β* for l-alanine and glycine (see Fig. 4), and these values were extrapolated to zero heating rate.

The melting temperature at zero heating rate takes into consideration the thermal lag^23,24^ and possible superheating.^23–25^ A good thermal contact between sample and sensor was provided by using silicon oil. This kept thermal lag (the slope of the lines in Fig. 4) small compared to the scatter of the *T*_fus_(*β*). Nevertheless, temperature correction due to thermal lag was taken into consideration and all temperatures were corrected accordingly. The thermodynamic melting temperatures of l-alanine and glycine extrapolated to zero heating rate were found to be *T*_fus_ = (608 ± 9) K and *T*_fus_ = (569 ± 7) K, respectively.

### Enthalpy of fusion of the amino acids

**Fig. 5:**
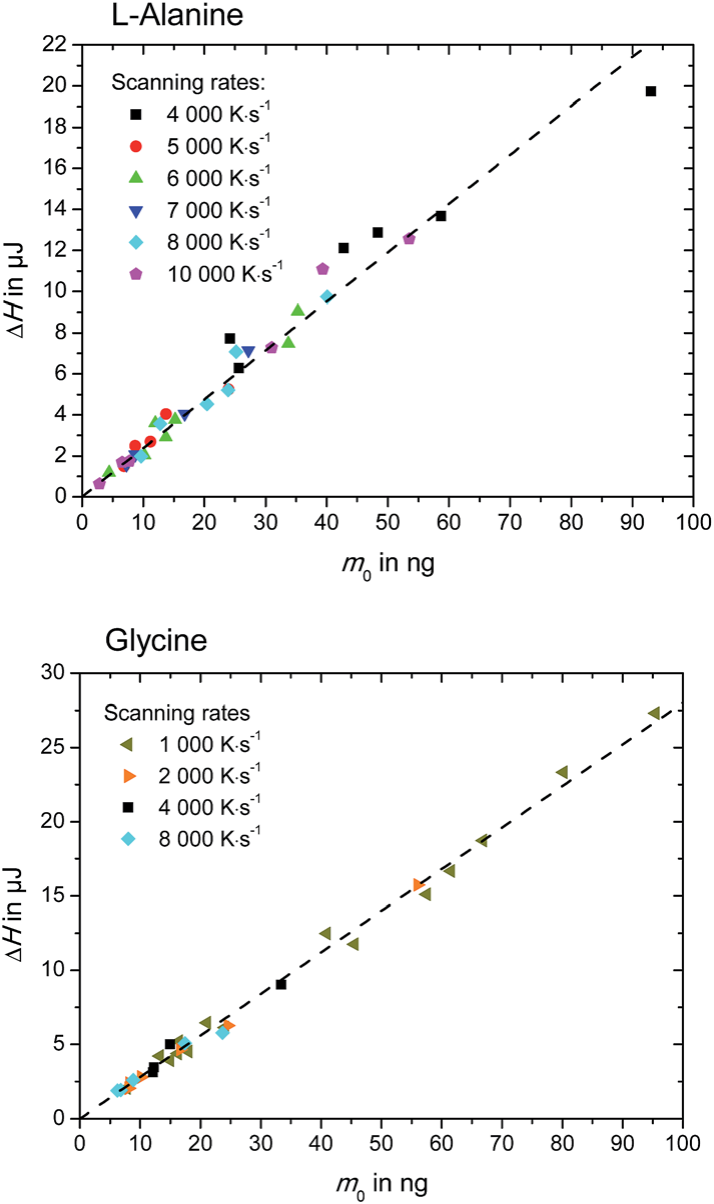
Enthalpy, Δ*H*, of l-alanine (upper figure) and glycine (lower figure), as determined in Fig. 3.

The enthalpy of fusion is defined as7
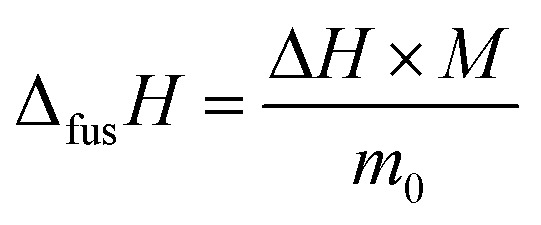
where the enthalpy, Δ*H* was determined as shown in Fig. 3 as area under the heat-flow curve. The molar mass of l-alanine and glycine is *M* = 89.1 g mol^−1^ and *M* = 75.1 g mol^−1^, respectively. As expected, the enthalpy, Δ*H*, depends linearly with the sample mass, *m*_0_, regardless of the scanning rates. This can be observed in Fig. 5. The slopes of the lines in Fig. 5 provide the specific enthalpies of fusion [J g^−1^].^12^ Finally, the entropy of fusion, Δ_fus_*S*, is determined as Δ_fus_*S* = Δ_fus_*H*/*T*_fus_. The obtained melting temperatures, enthalpies of fusion and entropies of fusion are listed in Table 2. The primary experimental data (45 measurements for l-alanine and 54 measurements for glycine) and the procedure of the uncertainty calculation are presented in ESI, Section S2.†

**Table 2:** FSC-measured melting properties

	*T*_fus_ in K	Δ_fus_*H* in kJ mol^−1^	Δ_fus_*S* in kJ K^−1^ mol^−1^
l-Alanine	608 ± 9	22 ± 5	0.036 ± 0.009
Glycine	569 ± 7	21 ± 4	0.037 ± 0.007

### Solubility predictions

In this work solubility of glycine and l-alanine in water were predicted with PC-SAFT using the melting properties from different sources and methods. The deviations between PC-SAFT predictions and experimental solubility data are quantified by the absolute relative deviations (ARD), which were calculated by:8
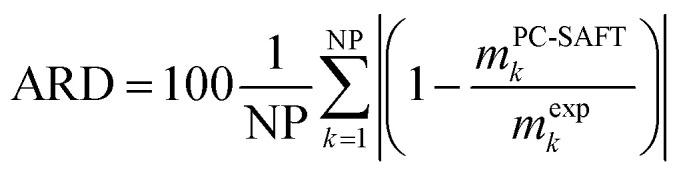
where *m*^PC-SAFT^ and *m*^exp^ are PC-SAFT predicted and experimental solubility of a maximum number of NP solubility data points.

### Solubility of glycine

**Fig. 6:**
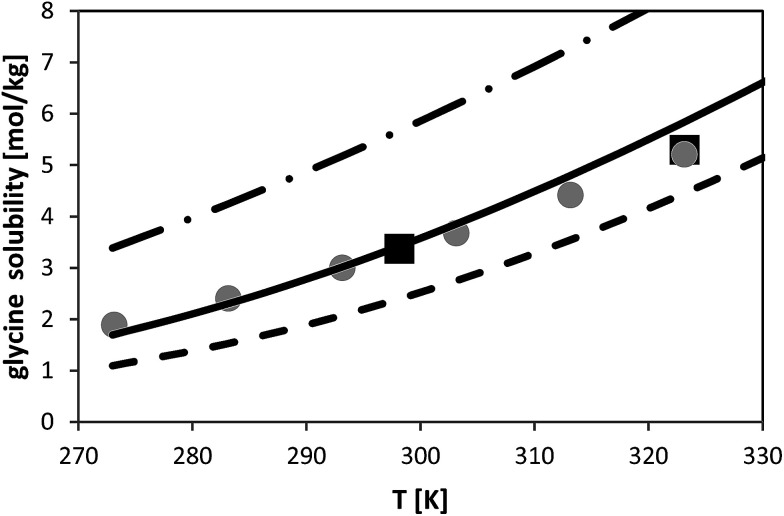
Glycine solubility in water as molality *vs.* temperature. Symbols represent experimental data (squares:^26^, circles:^27^). Lines represent PC-SAFT predictions with the parameters from Table 1 and FSC-measured melting properties from this work (full line) and with a 10% deviation from the FSC-measured value (dashed line: Δ_fus_*H* = 23.1 kJ mol^−1^; dashed-dotted line: Δ_fus_*H* = 18.9 kJ mol^−1^).

The temperature dependence of glycine solubility was measured in the literature and is well-known. Fig. 6 illustrates that the data in two chosen literature sources agree well with each other. The aim of this work was to use the FSC-measured melting properties of glycine listed in Table 2 in order to predict the solubility of glycine. The result of this prediction is illustrated in Fig. 6. It can be observed that PC-SAFT allows for quantitative predictions of the solubility behavior. Prediction means that all PC-SAFT parameters were fitted to solubility-independent data such as osmotic coefficients or mixture density.

In order to prove the required accuracy of experimentally measured melting properties, solubility was predicted with PC-SAFT using eqn (1) with the FSC-determined melting temperature (*T*_fus_ = 569 K) but with modified values for the enthalpy of fusion. The latter was modified in the range (Δ_fus_*H* − 10%) < Δ_fus_*H* < (Δ_fus_*H* + 10%), where Δ_fus_*H* means the FSC-measured value (Δ_fus_*H* = 21 kJ mol^−1^). The results are shown in Fig. 6; it can be observed that a deviation of 10% from the FSC-measured value for Δ_fus_*H* causes completely wrong solubility predictions. On the one hand, this points to the importance of experimentally-determined melting properties. On the other hand, these results illustrate that melting properties have to be known accurately, as only small inaccuracies in the melting properties might cause completely wrong solubility predictions.

### Solubility of l-alanine

**Fig. 7:**
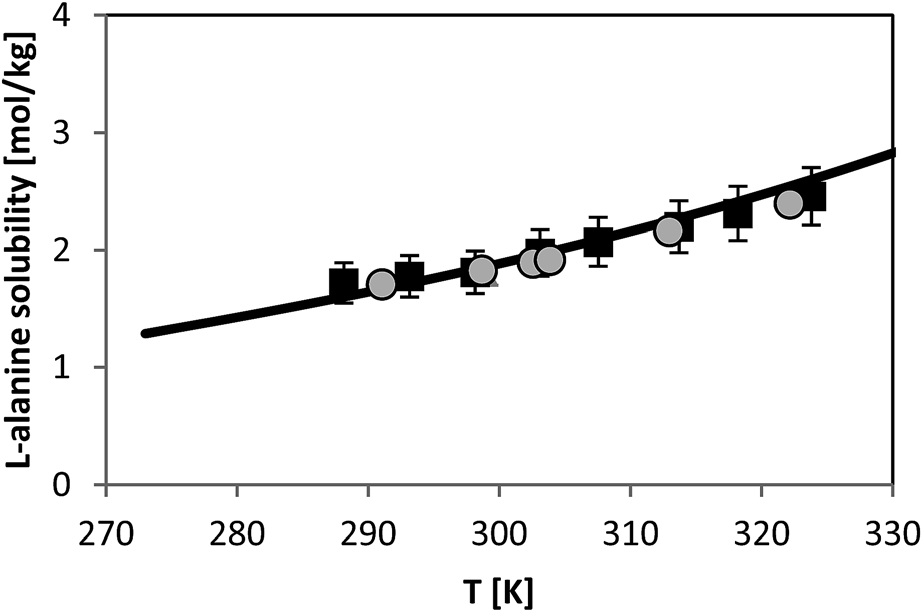
l-alanine solubility in water as molality *vs.* temperature. Symbols represent experimental data (squares:^28^, circles:^9^). Line represents PC-SAFT predictions with the parameters from Table 1 and FSC-measured melting temperature from this work, and a value for Δ_fus_*H* was adjusted to solubility at 298.15 K (Δ_fus_*H* = 23.7 kJ mol^−1^).

For l-alanine, much less experimental solubility data exist compared to glycine. In general, the solubility data have uncertainty of usually less than 3% from the absolute values. The temperature dependence of l-alanine solubility was measured in the literature and is presented in Fig. 7. For sake of overview two different literature sources are shown, those which are reliable based on our experience and on own (unpublished) measured values which we measured within the last decade. Fig. 7 illustrates that the data in two chosen literature sources^9,28^ agree well with each other. The aim of this work was to use the FSC-measured melting properties of l-alanine listed in Table 2, in order to predict the solubility of l-alanine. While the FSC-measured melting temperature was obtained very accurately, a rather high uncertainty was obtained for the value of Δ_fus_*H* of l-alanine, *i.e.* a value of Δ_fus_*H* = (22 ± 5) kJ mol^−1^ was measured. The use of this value caused inaccurate solubility predictions using PC-SAFT. Thus, this value was further adjusted by fitting it to the experimental solubility of l-alanine at 298.15 K from ref. 9 (1.828 mol l-alanine per kg water). The result of the modeling using this value is illustrated in Fig. 7. Consistently, the experimental value at 298.15 K was modeled accurately with PC-SAFT. Nevertheless, it can be also observed that PC-SAFT allows for quantitative predictions of the solubility behavior in the whole temperature range under consideration. Prediction means that all PC-SAFT parameters were fitted to solubility-independent data, such as osmotic coefficients or mixture density.

It should be mentioned that more quantitative modelling results can be achieved with PC-SAFT by re-adjusting the temperature-dependency of the *k*_*ij*_ parameter (given in footnote to Table 1 with a value of 2.91 × 10^−4^). This is typically done for solubility modelling with SAFT-based models.^29^ However, the motivation behind this work was to predict solubility of amino acids with thermodynamic models, in this work PC-SAFT, and to honestly state how reliable these predictions are if none of the used PC-SAFT parameters were fitted to any solubility data. Thus, the shown result has a predictive character at cost of lower accuracy compared to correlative modelling approaches.

### Comparison to literature values

The quantitative accuracy of the PC-SAFT predictions is a big advance compared to the classical way of thermodynamic solubility models for amino acids, in which usually the melting properties are freely fitted to experimental solubility data. Many examples can be found in the literature (see references in Table 3), in which either activity-coefficient models or equations of state were used to calculate activity coefficients for eqn (1) while fitting Δ_fus_*H* and *T*_fus_ to experimental solubility data. This procedure is still state-of-the art in the literature for components with inaccessible experimental melting properties. The fitted melting properties of glycine and l-alanine are summarized in Table 3, in which “method” denotes the kind of thermodynamic model used to fit the melting properties. It can be observed from Table 3 that the use of the activity-coefficient group-contribution model UNIFAC causes much too low values for Δ_fus_*H* and *T*_fus_ compared to FSC-measured data. Application of other g^E^ models (*e.g.* the lattice fluid theory NLF-HB) critically overestimates melting temperature. Neglecting activity coefficients in eqn (1) while fitting Δ_fus_*H* and *T*_fus_ to experimental solubility data causes even lower Δ_fus_*H* values. Applying SAFT or PC-SAFT to model activity coefficients and to fit these melting properties is apparently the less inaccurate method for fitting melting properties.

**Table 3:** Melting properties for glycine and l-alanine from literature (fitted to solubility data) or from this work (measured with FSC)

	*T*_fus_ [K]	Δ_fus_*H* [kJ mol^−1^]	Method	Source
**l** **-alanine**	**608**	**22**	**FSC**	**This work**
	**608**	**23.7**	**PC-SAFT**	**This work**
	539	9.24	UNIFAC	30
	1191	11.09	NLF-HB	31
	581.72	15.98	PC-SAFT	8
	692.4	21.1	PC-SAFT	9
**glycine**	**569**	**21**	**FSC**	**This work**
	**569**	**21.0**	**PC-SAFT**	**This work**
	433	7.36	UNIFAC	30
	535	3.5	Ideal solubility model	32
	714.3	17.54	PC-SAFT	9
	696	13.54	NLF-HB	31
	688	13.7	SAFT	7
	489	21.97	PC-SAFT	8

Thus, by comparing the values in Table 3 it becomes obvious that fitted melting properties using thermodynamic models can be any arbitrary values. This shows the urgent need for experimentally accessible melting properties, as these are the most reliable data. Using FSC yields accurate melting temperature while melting enthalpy is accompanied by a still relatively high uncertainty. Nevertheless, the reliability of the FSC-measured melting enthalpy is known.

The availability of direct experimental determination of melting properties of amino acids finally allows not only solubility predictions but even more quantifying activity coefficients of amino acids. These have not been accessible until now. Eqn (1) can thus be applied in order to calculate an “experimental” activity coefficient for amino acids in any mixture. For l-alanine + water mixture, the activity coefficient of l-alanine is found to be *γ*_alanine_(*x*^sat^ = 0.03188, *T* = 298.15 K) = 0.244 at the solubility of l-alanine in water at 298.15 K from ref. 9 using the FSC-measured melting properties. For glycine + water mixture, the activity coefficient of glycine is found to be *γ*_glycine_(*x*^sat^ = 0.05724, *T* = 298.15 K) = 0.31 at the solubility of glycine in water at 298.15 K from ref. 26. Access to such properties will further help developing more meaningful model parameters that will find broad acceptance in industry and academia.

## Conclusion

In this work for the first time, the thermodynamic parameters of melting for l-alanine and glycine were determined directly by using fast scanning calorimetry. The experimentally measured values were Δ_fus_*H* = (22 ± 5) kJ mol^−1^ and *T*_fus_ = (608 ± 9) K for l-alanine, and Δ_fus_*H* = (21 ± 4) kJ mol^−1^ and *T*_fus_ = (569 ± 7) K for glycine.

Based on these values solubility was predicted with PC-SAFT. Prior to the predictions, PC-SAFT parameters were fitted to solubility-independent thermodynamic properties such as osmotic coefficients or mixture densities. It was shown that this allowed accurately predicting l-alanine and glycine solubility in water over a broad temperature range. It should be noted that not only PC-SAFT but also other models can be used for such solubility predictions, and the combination of FSC with PC-SAFT is just one possibility. Certainly, PC-SAFT is among the most appropriate models for amino-acid solutions that exist in the literature.

The findings in this work will open the door for future in order to predict amino-acid solubility *in silico*.

## Conflicts of interest

There are no conflicts to declare.

## Supplementary Material

RA-008-C8RA00334C-s001
