# Supplementary material for: New experimental melting properties as access for predicting amino-acid solubility
Source: RSC Adv. 2018 Feb 8;8(12):6365–72. doi: 10.1039/c8ra00334c (PMC9078280; doi:10.1039/c8ra00334c)
Supplement: RA-008-C8RA00334C-s001 [file RA-008-C8RA00334C-s001.pdf]

Electronic Supplementary Material (ESI) for RSC Advances.
This journal is © The Royal Society of Chemistry 2018

SUPPLEMENTARY INFORMATION

Supplement to Manuscript ID.: RA-ART-01-2018-0003344

Title: New experimental melting properties as access for predicting amino-acid solubility

Author(s): Yeong Zen Chua, Hoang Tam Do, Christoph Schick, Dzmitry Zaitsau, Christoph Held

Section S1: Sample mass determination

Fast scanning calorimetry employs thin film chip sensors USF1, where a tiny sample was placed directly in the center of the measuring area of the sensor with hotspot of 500 μm diameter. The sample mass cannot be measured directly, but can be determined using two approaches, according to ref. [Cebe1]. The first approach for determination of sample mass is as ratio of the measured heat capacity increment, $\Delta C_p^{meas}(T_g)$ at the glass transition temperature, T_g, and the specific heat capacity increment, $\Delta c_p(T_g)$ at T_g. Unfortunately no specific heat capacity data at glass transition temperature is currently available. So this approach is not applicable in this work.

In the second approach, the sample mass is determined by the ratio of the measured heat capacity, $C_p(T)$, and specific heat capacity, $c_p(T)$, in the temperature range without mass loss due to sublimation or decomposition. Figure S1 shows an example of measurement scan used for sample mass determination for L-alanine in the temperature range 303 K to 473 K, where there is no sublimation and decomposition.

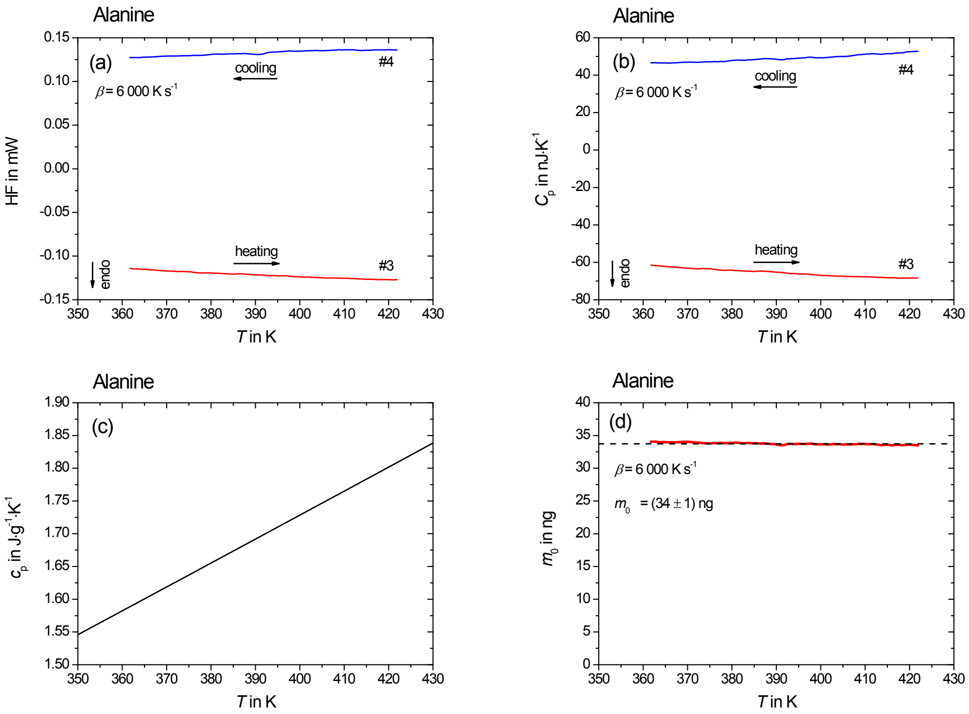

Figure S1: Evaluation of the measurement scans, heating step #3 (solid red line) and cooling step #4 (solid blue line), for the determination of sample mass. (a) Heat flow of the sample without silicon oil before melting, (b) heat capacity after correction with the symmetry line and empty measurement, (c) specific heat capacity of L-alanine [19], (d) resulting initial sample mass, m_0 (without silicon oil).

Section S2: Melting properties and uncertainty determination

The experimental melting temperatures, $T_{\text{fus}}(\beta)$ and enthalpy, ΔH, as function as scanning rate, β, as well as determined sample mass, m_0 and enthalpy of fusion, $\Delta_{\text{fus}}H$, for L-alanine and glycine are listed in Table S1 and Table S2, respectively. All the thermodynamic parameters were evaluated with an expanded uncertainty at a level of confidence of 95% and coverage factor, $k \approx 2$.

Temperature calibration was performed with recommended calibration materials (indium, tin, bismuth, lead and zinc) on the fast scanning calorimetry, Mettler Toledo Flash DSC1. From the temperature calibration, the temperature corrections for L-alanine and glycine are $\delta T = 8$ K and $\delta T = 6$ K, respectively. The temperature corrections, δT is employed for extrapolated peak onset temperature, $T_{\text{fus}}(\beta)$ for L-alanine and glycine. The thermodynamic melting temperature is determined as $T_{\text{fus}} = T_{\text{fus}}(\beta \rightarrow 0)$, [22] with a linear fit, as shown in Figure 4. From the linear fit, the errors for L-alanine and glycine are $\varepsilon = 4$ K and $\varepsilon = 1$ K. The uncertainty of melting temperature, T_{fus}, is determined as

$$\delta T_{\text{fus}} = \sqrt{\varepsilon^2 + \delta T^2} \qquad \text{(S1)}$$

The thermodynamic melting temperatures with uncertainly are $T_{\text{fus}} = (608 \pm 9)$ K for L-alanine and $T_{\text{fus}} = (569 \pm 7)$ K for glycine.

The enthalpy of fusion for L-alanine and glycine are determined from the average of the results in Table S1 and Table S2, as $\Delta_{\text{fus}}H = (22 \pm 5)$ kJ·mol^{-1} and $\Delta_{\text{fus}}H = (21 \pm 4)$ kJ·mol^{-1}. The entropy of fusion is determined as $\Delta_{\text{fus}}S = \Delta_{\text{fus}}H \,/\, T_{\text{fus}}$. The uncertainly of the entropy of fusion is calculated as

$$\delta\left(\Delta_{\mathrm{fus}}S\right)=\left[\frac{\delta(\Delta_{\mathrm{fus}}H)}{T_{\mathrm{fus}}}\right]+\left[\frac{\Delta_{\mathrm{fus}}H}{\left(T_{\mathrm{fus}}\right)^{2}}\cdot\delta T_{\mathrm{fus}}\right] \qquad \text{(S2)}$$

The entropy of fusion for L-alanine and glycine are $\Delta_{\mathrm{fus}}S = (0.036 \pm 0.009)$ kJ·K^{-1}·mol^{-1} and $\Delta_{\mathrm{fus}}S = (0.036 \pm 0.009)$ kJ·K^{-1}·mol^{-1}, respectively.

Table S1: Experimental melting temperature, $T_{\mathrm{fus}}(\beta)$, and enthalpy, ΔH, as well as determined sample mass, m_0 and enthalpy of fusion, $\Delta_{\mathrm{fus}}H$ for L-alanine.

Exp. No.	β in K·s^{-1}	m_0 in ng	$T_{\mathrm{fus}}(\beta)$ in K	ΔH in µJ	$\Delta_{\mathrm{fus}}H$ in kJ·mol^{-1}
1	1 000	5 ± 1	601 ± 8	2	24
2	1 000	69 ± 1	608 ± 8	20	27
3	2 000	43 ± 1	607 ± 8	10	21
4	2 000	48 ± 1	610 ± 8	9	16
5	2 000	94 ± 1	608 ± 8	18	17
6	2 000	52 ± 1	609 ± 8	11	18
7	2 000	67 ± 1	608 ± 8	18	24
8	2 000	76 ± 1	609 ± 8	20	24
9	2 000	45 ± 1	608 ± 8	12	23
10	2 000	56 ± 1	608 ± 8	20	31
11	2 000	84 ± 1	610 ± 8	22	23
12	2 000	52 ± 1	611 ± 8	18	30
13	4 000	26 ± 1	609 ± 8	7	22
14	4 000	43 ± 1	620 ± 8	13	26
15	4 000	94 ± 2	622 ± 8	20	19
16	4 000	49 ± 1	615 ± 8	13	24
17	4 000	59 ± 1	607 ± 8	14	21
18	5 000	7 ± 1	601 ± 8	2	20
19	5 000	9 ± 1	604 ± 8	3	26
20	5 000	24 ± 1	605 ± 8	6	20
21	5 000	12 ± 1	605 ± 8	3	22
22	6 000	5 ± 1	605 ± 8	2	24
23	6 000	34 ± 1	617 ± 8	8	20
24	6 000	11 ± 1	614 ± 8	3	18
25	6 000	12 ± 1	606 ± 8	4	27
26	6 000	36 ± 1	614 ± 8	10	23
27	6 000	16 ± 1	604 ± 8	4	23

28	6 000	14 ± 1	603 ± 8	3	19
29	7 000	9 ± 1	617 ± 8	3	22
30	7 000	8 ± 1	603 ± 8	2	20
31	7 000	28 ± 1	615 ± 8	8	24
32	7 000	17 ± 1	613 ± 8	5	22
33	8 000	12.8 ± 1	614 ± 8	4	25
34	8 000	25.2 ± 2	614 ± 8	8	25
35	8 000	117.8 ± 1	618 ± 8	28	21
36	8 000	40.1 ± 1	614 ± 8	10	22
37	8 000	23.9 ± 1	615 ± 8	6	20
38	8 000	20.5 ± 1	619 ± 8	5	20
39	8 000	9.7 ± 1	614 ± 8	2	19
40	10 000	53.5 ± 1	623 ± 8	13	21
41	10 000	31.1 ± 1	614 ± 8	8	21
42	10 000	39.4 ± 1	603 ± 8	12	26
43	10 000	2.9 ± 1	603 ± 8	7	20
44	10 000	7.8 ± 1	616 ± 8	2	21
45	10 000	6.6 ± 1	609 ± 8	2	23

Table S2: Experimental melting temperature, $T_{fus}(\beta)$, and enthalpy, ΔH, as well as determined sample mass, m_0 and enthalpy of fusion, $\Delta_{fus}H$ for glycine.

Exp. No.	β in K·s^{-1}	m_0 in ng	$T_{fus}(\beta)$ in K	ΔH in µJ	$\Delta_{fus}H$ in kJ·mol^{-1}
1*	1 000		570 ± 6		
2*	1 000		569 ± 6		
3*	1 000		569 ± 6		
4*	1 000		569 ± 6		
5*	1 000		569 ± 6		
6*	1 000		568 ± 6		
7*	1 000		571 ± 6		
8*	1 000		570 ± 6		
9*	1 000		569 ± 6		
10*	1 000		569 ± 6		
11*	1 000		570 ± 6		
12	1 000	18 ± 1	570 ± 6	5	19
13	1 000	14 ± 1	571 ± 6	5	24

14	1 000	17 ± 1	569 ± 6	5	21
15	1 000	81 ± 13	571 ± 6	24	22
16	1 000	96 ± 25	571 ± 6	28	22
17	1 000	67 ± 8	570 ± 6	19	22
18	1 000	22 ± 1	568 ± 6	7	24
19	1 000	8 ± 1	568 ± 6	3	21
20	1 000	62 ± 6	571 ± 6	17	21
21	1 000	46 ± 2	567 ± 6	12	20
22	1 000	58 ± 1	567 ± 6	16	20
23	1 000	41 ± 1	567 ± 6	13	23
24	1 000	15 ± 1	567 ± 6	4	20
25	2 000	11 ± 1	570 ± 6	3	21
26	2 000	17 ± 1	572 ± 6	5	21
27	2 000	9 ± 1	572 ± 6	3	19
28	2 000	25 ± 2	570 ± 6	7	20
29	2 000	9 ± 1	571 ± 6	3	22
30	2 000	57 ± 8	571 ± 6	16	22
31	4 000	13 ± 1	572 ± 6	4	21
32	4 000	13 ± 1	572 ± 6	4	20
33	4 000	15 ± 1	570 ± 6	5	26
34	4 000	34 ± 2	573 ± 6	10	21
35*	6 000		574 ± 6		
36*	6 000		571 ± 6		
37*	6 000		572 ± 6		
38*	6 000		572 ± 6		
39*	6 000		574 ± 6		
40	8 000	7 ± 1	572 ± 6	2	22
41	8 000	18 ± 1	572 ± 6	6	22
42	8 000	9 ± 1	572 ± 6	3	22
43	8 000	7 ± 2	572 ± 6	2	23
44	8 000	24 ± 1	573 ± 6	6	19
45*	10 000		573 ± 6		
46*	10 000		575 ± 6		
47*	10 000		578 ± 6		
48*	10 000		574 ± 6		

49*	10 000		572 ± 6		
50*	10 000		572 ± 6		
51*	10 000		572 ± 6		
52*	10 000		577 ± 6		
53*	10 000		572 ± 6		
54*	10 000		574 ± 6		

*No initial sample mass without silicon oil is determined. Silicon oil is directly added to sample to improve the thermal contact while obtaining the $T_{\mathrm{fus}}(\beta)$.

[19: J. O. Hutchens, A.G. Cole and J. W. Stout, Journal of American Chemical Society, 1960, 82, 4813-4815.]

[22: G. W. H. Hohne, H. K. Cammenga, W. Eysel, E. Gmelin and W. Hemminger, Thermochim. Acta, 1990, 160, 1-12]

[Cebe1: P. Cebe, B. P. Partlow, D. L. Kaplan, A. Wurm, E. Zhuravlev, C. Schick, Thermoch. Acta, 2015, 615, 8-14]
